# Editorial: Still Searching for the Origin of Migraine: From Comorbidities to Chronicization

**DOI:** 10.3389/fnhum.2022.880365

**Published:** 2022-04-01

**Authors:** Gianluca Coppola, Roberta Messina, Linxin Li, Claudia Altamura

**Affiliations:** ^1^Department of Medical-Surgical Sciences and Biotechnologies, Sapienza University of Rome, Latina, Italy; ^2^Neuroimaging Research Unit and Neurology Unit, IRCCS San Raffaele Scientific Institute, Milan, Italy; ^3^Department of Clinical Neuroscience, University of Oxford, Oxford, United Kingdom; ^4^Headache and Neurosonology Unit, Fondazione Policlinico Universitario Campus Bio-Medico, Rome, Italy

**Keywords:** migraine, comorbid, chronic, physiopathologic mechanism, progression

Migraine is a multifactorial disorder with huge ramifications in the central nervous system. Despite the enormous progress made in recent years in understanding the pathophysiological mechanisms underlying this painful condition, little is known about the factors behind the evolution from the episodic to the chronic form of migraine. One of the main factors subtending this transformation is undoubtedly the excessive, even compulsive, use of symptomatic drugs, whatever they may be. Migliore et al. have shown how complicated the psychopathological profile of medication-overuse headache (MOH) patients is. They are unable to regulate and properly recognize their emotions and are more anxious and depressed. This study also observed that this psychopathological profile influences the severity and impact of the disease in daily life. The tendency to overuse symptomatic drugs most probably has also a genetic basis. The worframin gene (His611Arg) influences drug consumption in psychiatric patients with impulsive addictive behavior. Di Lorenzo et al. observed that the His611Arg polymorphism led to increased use of symptomatic drugs only in patients with MOH, especially those using combination drugs compared with non-medication over-users. Moavero et al. also investigated MOH in a group of children and adolescents with migraine and found that not all patients benefit from drug withdrawal. This finding questions that not all patients with migraine overuse can be correctly classified according to the MOH criteria in the second and third revision of the International Classification of Headache Disorders. These results underline once again how evolving the diagnostic criteria for chronic migraine and its secondary counterpart MOH still are. Nevertheless, these data may indicate the existence of a genetic predisposition underlying the response to withdrawal, a subject not yet investigated.

The existence of episodic syndromes associated with migraine in childhood and adolescence supports the idea that genes play an important additive role in the clinical manifestation of the disease. Cyclic vomiting, an undoubtedly under-diagnosed and very disabling syndrome, about which little is known from a pathophysiological point of view, represents an example. Raucci et al. did a great job in putting together a task force of experts from different disciplines with the valuable aim of reviewing the known salient data on the disease and proposing future research directions.

A child's migraine is undoubtedly an excellent model for studying migraine pathology when it is still in its infancy, i.e., when the pathophysiological mechanisms that abnormally regulate the CNS excitability of the young migraine patient begin to show the first signs of themselves. Rho et al. retrospectively evaluated the EEG of children with different headache types and found a higher frequency of rhythm abnormalities in patients with migraine with aura. Their data also suggest that these patients can experience higher levels of disability in daily life. In general, this retrospective study underlines that migraine belongs to central nervous system disorders characterized by cortical dysrhythmia such as epilepsy, an accessual pathology with which migraine is likely to share disease mechanisms. They likely have at least a partial genetic structure in common, as underlined by the most recent genome-wide association studies that have identified a number of gene loci associated with migraine risk, such as those involved in synaptic plasticity, glutamate homeostasis, pain-related pathways, vascular regulation, and vascular tissue. This gene set-up could explain the cardiovascular comorbidity found in particular in migraine with aura patients, in whom patency of the interatrial septum is most frequently seen and whose need for closure is still debated. This is based on the hypothesis that if the patency is broad enough, it may favor the passage of paradoxical embolisms that could trigger cortical spreading depression, the electrocortical phenomenon believed to determine the aura phenomenon. This correlation between patent foramen ovale and migraine is precisely what Liu et al. discuss.

Various disorders may be present in comorbidity with migraine and thus place an additional burden on the patient's shoulders. Anxiety disorder is one of the most frequently detected in studies on the subject, reviewed by Karimi et al. Anxiety disorders are often associated with mood disorders, chronic fatigue, and fibromyalgia, which are again comorbid with migraine, especially when it is chronic (see Karsan and Goadsby). Underlying these disorders may be a shared pro-algogenic terrain, also be favored by the low levels of vitamin D found by Rebecchi et al., especially in patients with chronic migraine. The vitamin D level in the blood correlates with the individual's circadian rhythm. In an fMRI study, Baksa et al. found that different circadian peaks of migraine attack onset are associated with different interictal brain activity in response to threatening fearful stimuli. It is difficult to say whether the same variations could be detected in migraine patients with aura, in whom Carvalho et al. found a delayed motor control response and instability under conditions of external perturbation. The execution of adequate corrective motor responses requires adequate integration between the neural, sensory and musculoskeletal systems. Their dysfunction leads to abnormalities of multiple functional levels in the central nervous system, underlining the extensive central nervous system ramification of migraine pathology.

In conclusion, considering the plethora of pathologies with which migraine, especially when it evolves into a chronic form, is comorbid (see Altamura et al.), we think it is time to move from the original definition of migraine as a “*disease*” to the definition of a “*migraine syndrome*,” which incorporates both the pain manifestation and the parade of central and systemic symptoms and pathologies with which it shares comorbidity. In this light, a multidisciplinary approach is necessary to obtain a proficient and persistent relief of pain and other symptoms in people with migraine.

## Author Contributions

GC drafted the manuscript. CA, RM, and LL reviewed it for intellectual content. All authors contributed to the article and approved the submitted version.

## Conflict of Interest

The authors declare that the research was conducted in the absence of any commercial or financial relationships that could be construed as a potential conflict of interest.

## Publisher's Note

All claims expressed in this article are solely those of the authors and do not necessarily represent those of their affiliated organizations, or those of the publisher, the editors and the reviewers. Any product that may be evaluated in this article, or claim that may be made by its manufacturer, is not guaranteed or endorsed by the publisher.

